# Host-Mediated Selection Shapes Conserved Root Bacterial Microbiomes Across Geographically Separated *Thismia* Species

**DOI:** 10.3390/plants15091316

**Published:** 2026-04-25

**Authors:** Phuwadon Udompongpaiboon, Nuttapol Noirungsee, Sahassawat Chailungka, Ponsit Sathapondecha, Sahut Chantanaorrapint, Lompong Klinnawee

**Affiliations:** 1 Division of Biological Science, Faculty of Science, Prince of Songkla University, Songkhla 90110, Thailand; phuwadon.u@gmail.com (P.U.); ponsit.s@psu.ac.th (P.S.); sahut.c@psu.ac.th (S.C.); 2 Department of Biology, Faculty of Science, Chiang Mai University, Chiang Mai 50200, Thailand; nuttapol.n@cmu.ac.th (N.N.); sahassawat6811@gmail.com (S.C.); 3 Center of Excellence in Microbial Diversity and Sustainable Utilization, Chiang Mai University, Chiang Mai 50200, Thailand

**Keywords:** *Thismia*, mycoheterotrophic plant, endophytic bacteria, bacteroidota, nitrogen fixation, host-mediated selection

## Abstract

*Thismia* species are non-photosynthetic plants entirely dependent on fungal partners for carbon and nutrients. While their arbuscular mycorrhizal associations are well-documented, bacterial symbiont roles remain unexplored. Using 16S rRNA gene amplicon sequencing, we investigated endophytic bacterial communities in *T. gardneriana*, *T. javanica*, and *T. mirabilis* from geographically distinct locations in Thailand. Despite geographic separation, *Thismia* spp. consistently harbored bacterial compositions taxonomically and functionally distinct from surrounding soil microbiomes. Root endospheres were significantly enriched in Pseudomonadota and Bacteroidota, particularly *Puia*, while showing reduced compositional dynamics of Acidobacteriota and Planctomycetota. Bacterial communities in *Thismia* roots were markedly distinct from surrounding soil, while root endosphere communities from geographically distinct habitats clustered together regardless of spatial separation. Mantel and partial Mantel tests confirmed that host species identity, not geographical location, was the primary predictor of root bacterial community structure. Functional prediction analyses suggested root-associated communities were enriched for nitrogen cycling pathways, particularly nitrogen fixation and nitrate reduction. The selective enrichment of Bacteroidota, known for nitrogen fixation and phosphate mobilization, suggests these bacteria provide critical nutritional support in nutrient-poor forest floor environments. Isolated root strains belonged exclusively to Bacillota, including *Neobacillus* with plant growth-promoting traits. Our findings highlight the importance of tripartite plant–fungal–bacterial interactions in *Thismia* nutritional ecology.

## 1. Introduction

*Thismia* species are achlorophyllous, mycoheterotrophic plants (MHPs) that completely lack the ability to photosynthesize. The morphological and molecular evidence shows them to be entirely dependent on arbuscular mycorrhizal fungi (AMF) networks for their carbon supply and all organic nutrients [1,2,3]. MHPs follow a more restricted association with their AMF partners in terms of phylogenetic diversity when compared with surrounding green plants, targeting more clustered lineages of AMF, independent of geographic origin [4]. Ya, et al. (2024) [5] showed that AMF from the Glomeraceae family that were associated with *Thismia* belonged to only one subclade, while green plants formed mutualistic associations with broader subclades of AMF. A study in temperate Australia and New Zealand has shown that *Thismia* species are dependent on AMF with considerable specificity towards a narrow range of Glomeraceae [6]. Furthermore, highly specific and phylogenetically conserved AMF interactions have been documented in previous studies of the Thismiaceae [3,6]. In addition to their critical dependence on AMF, *Thismia* species may also require endophytic bacteria for their growth and survival, as recently demonstrated for *T. gardneriana* in a lowland tropical rainforest in Thailand [3].

A study of the MHP *Pterospora andromedea* demonstrated that it could shape bacterial communities through its roots. Different root structures, including non-mycorrhizal and ectomycorrhizal roots, were colonized by distinct compositions of endophytic bacteria that differed from those found in the rhizosphere and bulk soil. *P. andromedae* selectively enriched Burkholderiales in its roots [7]. These bacteria have been documented as nitrogen-fixing endophytic bacteria in several plant species and have been identified as the most common endosymbiotic bacteria in fungal hyphae. They are found in both AMF and ectomycorrhizal fungi (EcMF) [8,9,10]. Similarly, *Gastrodia elata* growing naturally in a forest in China was shown to consistently recruit Mucomycota while excluding Acidobacteria and Chloroflexi from its rhizosphere compared with the surrounding bulk soil [11]. This selective recruitment of beneficial microorganisms is further exemplified in the MHP orchid *Arachnitis uniflora*. This fully MHP species harbors diverse plant growth-promoting bacteria (PGPB) such as *Bacillus megaterium*, *Paenibacillus* spp. and *Chryseobacterium* spp. in both its rhizosphere and endosphere. The plant growth-promoting traits exhibited by these bacteria include phosphate solubilization, indole acetic acid production and siderophore production. Notably, these bacterial isolates also possess antagonistic effects against potential plant pathogens, suggesting that MHPs may selectively maintain beneficial bacterial communities that both promote growth and provide protection against plant pathogens [12].

The involvement of bacteria in nutrient acquisition is expected to be critical for *Thismia* species, as their extremely reduced and simplified root systems limit their ability to access soil nutrients directly [13,14]. Therefore, they likely depend on endophytic bacteria to improve nutrient uptake efficiency, especially for nitrogen and phosphorus, which are typically scarce in tropical ecosystem soils [15,16,17]. However, tropical forest soils and litter layers contain an abundance of PGPB that can facilitate enhanced nutrient uptake by plants. In tropical forests, several microorganisms are involved in the nitrogen cycle, including nitrogen-fixing bacteria, ammonia-oxidizing bacteria and archaea, heterotrophic nitrifying microorganisms, and anammox bacteria, as well as denitrifying bacteria [16]. Forest soils also contain phosphorus-, potassium-, and silicon-solubilizing bacteria, which can mobilize soil minerals through organic acid production and pH modification to promote plant growth by increasing the availability of soil nutrients [18]. Therefore, *Thismia* species with their reduced root system would partially benefit from such bacterial partnerships for nutrient acquisition.

The tropical rainforests of Thailand, particularly across the Thai Peninsula, represent one of the species diversity centers for the genus *Thismia* with the region’s high humidity, dense canopy, and varied lowland forest ecosystems [13]. These shade-dependent MHPs inhabit the forest understory, where they prefer environments with abundant leaf litter, high humidity, and low-light conditions [19,20]. Our previous studies have documented several endemic *Thismia* species in Thailand, including *T. angustimitra*, *T. brunneomitroides*, *T. claviformis*, *T. clavigeroides, T. filiformis, T. mirabilis, T. nigricans*, *T. submucronata*, and *T. thaithomgiana.* In addition to these endemic taxa, more widely distributed species such as *T. arachnites*, *T. gardneriana*, and *T. javanica* are also present in the region [13,14,21,22,23,24,25,26,27]. However, field surveys remain challenging due to their predominantly belowground life cycle. *Thismia* plants typically remain dormant beneath the soil surface, emerging only briefly during flowering periods. Moreover, the occurrence of these plants in their habitats may be inconsistent and unpredictable across years, with populations appearing sporadically depending on environmental conditions. While associations between *Thismia* and AMF have been documented, the bacterial communities harbored by these enigmatic plants remain unexplored. We therefore investigated the conservation biogeography of endophytic bacterial communities and their diversity in three *Thismia* species across geographically distinct locations in Thailand, hypothesizing that despite biological and geographic independence, *Thismia* species harbor conserved core bacterial endophytic communities, reflecting their specialized ecological niche.

## 2. Results

### 2.1. Comparison of Bacterial Composition Between the Endosphere and Soil of Thismia Species

**Figure 1:**
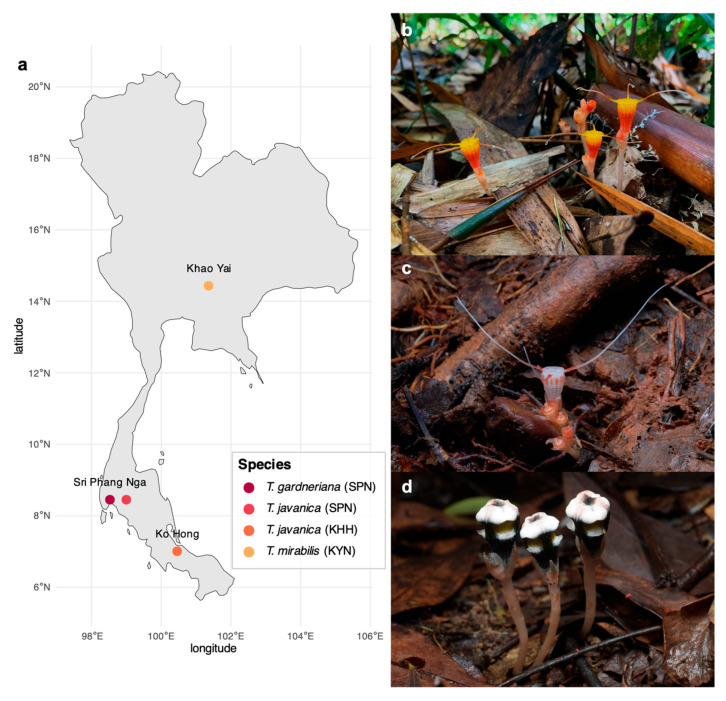
Geographic distribution of *Thismia* species collected in this study. The map shows the sampling locations in 2023 of three *Thismia* species across diverse ecosystems in Thailand (**a**). *T. gardneriana* (**b**) was collected from May to December in Si Phang Nga National Park (SPN) in a lowland tropical rain forest in the southern Peninsula. *T. javanica* (**c**) was collected from SPN from May to December, and from Ko Hong Hill (KHH) further south in August. *T. mirabilis* (**d**) was collected in June from Khao Yai National Park (KYN) in central Thailand. All the *Thismia* species emerged from leaf litter and grew amidst decomposing organic matter, illustrating the typical mycoheterotrophic habitat of these understory plants.

Three *Thismia* species were sampled from three geographically distinct locations across Thailand (Figure 1a), reflecting the isolated habitats of these MHPs across the Thai Peninsula. Field observations revealed that all three *Thismia* species inhabit the forest floor in areas with abundant leaf litter and decaying plant material (Figure 1b–d). The MHPs were found exclusively in undisturbed forest ecosystems during the wet season, when precipitation and humidity levels were high but temperatures were low (Supplementary Figure S1), suggesting specific microclimatic requirements.

**Figure 2:**
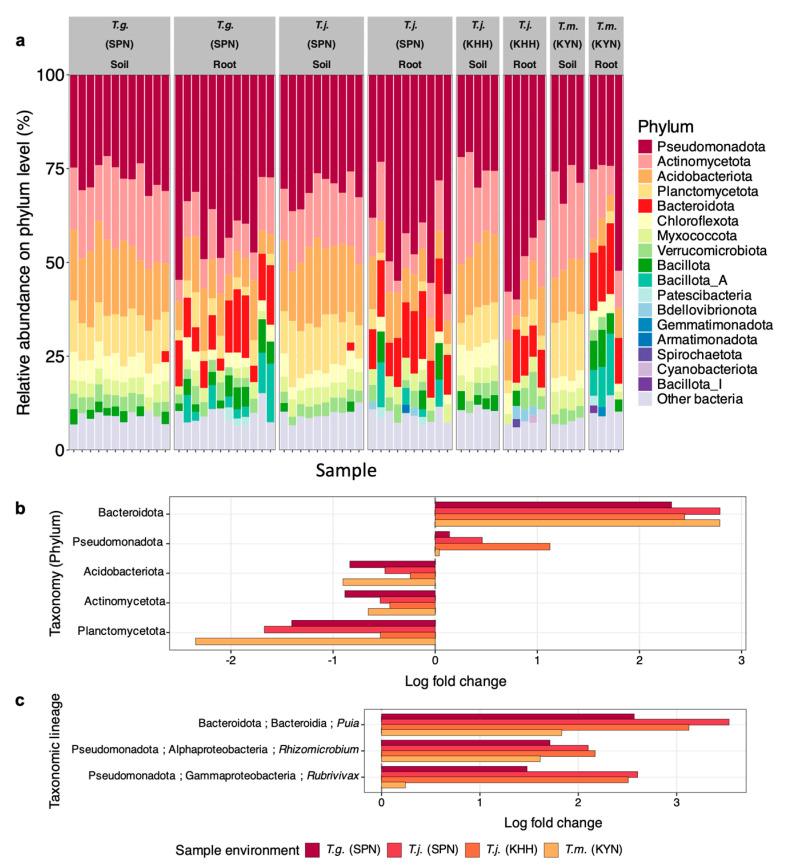
Taxonomic profiles reveal conserved bacterial compositions in *Thismia* species across geographic locations. (**a**) Relative abundances, expressed as the proportion of each bacterial taxon relative to the total number of classified sequences per sample (%), of bacterial phyla in soil and root samples from *T. gardneriana* (*T.g.*) collected at Si Phang Nga National Park (SPN) (*n* = 12), *T. javanica* (*T.j.*) from SPN (*n* = 10) and Ko Hong Hill (KHH) (*n* = 5), and *T. mirabilis* (*T.m.*) from Khao Yai National Park (KYN) (*n* = 4). Note the consistent enrichment of Bacteroidota (red) in root tissues compared with adjacent soil samples, regardless of host species or geographic origin. Phyla with less than 2.00% abundance were grouped as “Other bacteria”. Differentially abundant bacterial taxa between soil and root compartments identified by ANCOM-BC, shown at phylum (**b**) and genus level within full taxonomic lineage (**c**). Log fold change (LFC) values represent enrichment or depletion of taxa in the root relative to surrounding soil; positive values indicate enrichment and negative values indicate depletion. Only taxa passing the significance threshold (*p* < 0.001) are displayed: at the phylum level, all significantly differentially abundant phyla showing consistent directionality across all sampling groups are shown; at the genus level, only genera within Bacteroidota and Pseudomonadota showing consistent directionality across all sampling groups are displayed.

The amplicon sequencing of 16S rRNA gene of bacteria associated with the three *Thismia* species revealed distinct taxonomic profiles between roots and surrounding soil, suggesting selective bacterial recruitment by these MHPs (Figure 2). At the phylum level (Figure 2a), regardless of species, Pseudomonadota dominated across all root samples (31.44–45.71%) while showing lower relative abundance in corresponding soil samples (27.31–28.71%). This enrichment pattern was consistent across all three species, indicating a conserved association. Actinomycetota and Acidobacteriota exhibited higher abundance in soil than root tissues, suggesting these taxa were reduced from the endophytic compartment. Moreover, Bacteroidota showed substantial and consistent enrichment in root tissues of all three *Thismia* species. In *T. gardneriana* roots, Bacteroidota constituted approximately 10.76% of the bacterial composition, representing a 3.67-fold increase compared with adjacent soil samples (2.93%). In *T. javanica* roots, Bacteroidota constituted 12.89% of the bacterial composition, a 5.93-fold enrichment over the corresponding soil (2.17%). This pattern continued in *T. mirabilis* roots, where Bacteroidota comprised 15.38% of bacterial composition versus less than 2% in the surrounding soil.

To confirm the observed compositional differences, we performed ANCOM-BC differential abundance analysis comparing root and bulk soil compartments across all four sampling groups. At the phylum level, Bacteroidota and Pseudomonadota were significantly enriched in root tissues relative to soil, consistent with the patterns observed in relative abundance profiles. Conversely, Planctomycetota, Acidobacteriota, and Actinomycetota were significantly depleted in root (Figure 2b). At the genus level, *Puia* within the class Bacteroidia of the phylum Bacteroidota showed the strongest and most consistent enrichment in root tissues across all sampling groups, followed by *Rhizomicrobium* within the class Alphaproteobacteria and *Rubrivivax* within the class Gammaproteobacteria, both belonging to the phylum Pseudomonadota (Figure 2c). These results statistically validate the selective enrichment and depletion patterns described above, confirming that the observed taxonomic shifts represent significant host-mediated filtering rather than stochastic variation.

### 2.2. The Bacterial Diversity and Community in the Endosphere of Thismia Species

**Figure 3:**
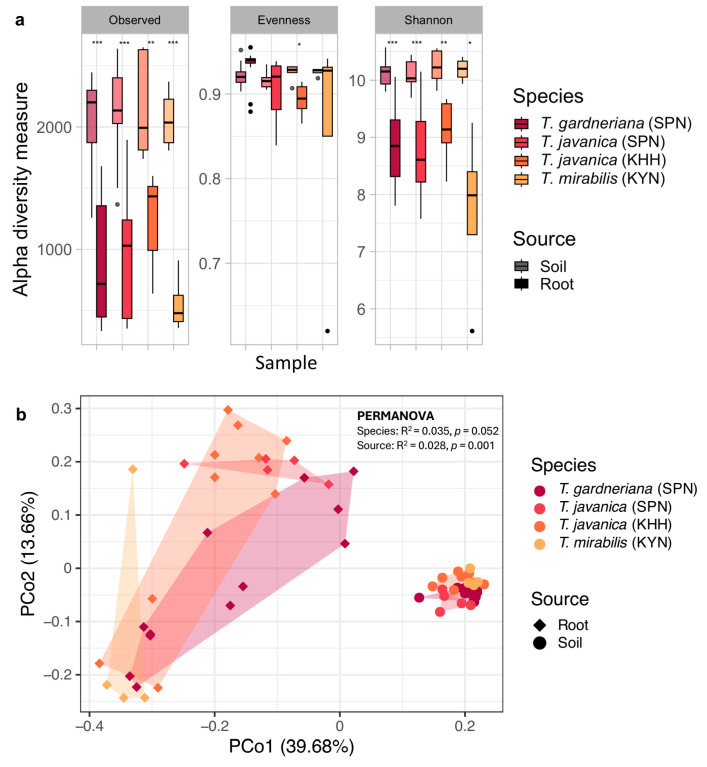
Bacterial diversity and community structures in the soil and root endosphere of three *Thismia* species across geographically distinct sites. Alpha diversity metrics, including observed ASV richness, evenness, and Shannon diversity indexes, were determined for *T. gardneriana* from Si Phang Nga National Park (SPN) (*n* = 12), *T. javanica* from SPN (*n* = 10) and Ko Hong Hill (KHH) (*n* = 5), and *T. mirabilis* from Khao Yai National Park (KYN) (*n* = 4) (**a**). The x-axis represents sample groups denoted by species and sampling location. Within each panel, sample source (soil vs. root) is distinguished by shading, with darker shading representing root samples and lighter shading representing soil samples. Statistical analysis was performed using the Wilcoxon rank sum test with asterisks indicating significant differences (* *p* ≤ 0.05, ** *p* ≤ 0.01, *** *p* ≤ 0.001) between soil and root compartments. Principal coordinate analysis (PCoA) based on Bray–Curtis dissimilarity revealed distinct clustering of bacterial communities by sample source (soil vs. root) rather than by species or geographic location (**b**). Sample source is indicated by symbol shape, with circles representing root samples and diamonds representing soil samples.

Alpha diversity analysis based on bacterial amplicon sequence variants (ASVs) revealed substantial differences in bacterial community structure between root tissues and surrounding soil across all three *Thismia* species (Figure 3a). Soil samples consistently harbored significantly higher bacterial diversity compared to corresponding root samples, indicating strong selective filtering of the soil microbiota by *Thismia*. The observed ASV richness in soil samples reached approximately 2000 unique sequences across all three *Thismia* species, reflecting the inherently high diversity of tropical forest soils. In contrast, root samples exhibited dramatically reduced ASV counts. However, evenness remained relatively consistent between soil and root environments. The Shannon diversity indexes, which account for both richness and evenness, further confirmed the significantly lower bacterial diversity within the *Thismia* root tissues (Figure 3a).

To assess whether bacterial community structure differed between soil and root compartments and to evaluate the relative contributions of sample source and host species to community variation, Principal Coordinate Analysis (PCoA) based on Bray–Curtis dissimilarity was performed on combined soil and root samples. The analysis revealed that bacterial community clustering was source-dependent (Figure 3b). PCo1, representing the axis of greatest variation, and PCo2, representing the second-largest orthogonal source of variation, explained 39.68% and 13.66% of the total variation, respectively. PERMANOVA analysis confirmed a significant effect of sample source on bacterial community structure (R^2^ = 0.035, *p* = 0.001), while the host species effect approached significance (R^2^ = 0.028, *p* = 0.052). To further examine bacterial community variation within each compartment independently, PCoA was performed separately for soil and root samples (Supplementary Figure S3a,b). For soil samples, PCo1 and PCo2 explained 18.59% and 16.79% of total variation, respectively, and PERMANOVA confirmed that geographic location had a significant effect on soil bacterial community structure (R^2^ = 0.218, *p* = 0.001), indicating that sampling site was a primary driver of soil microbial variation. For root samples, PCo1 and PCo2 explained 29.91% and 15.41% of total variation, respectively, and PERMANOVA revealed that host species had a significant effect on root bacterial community structure (R^2^ = 0.207, *p* = 0.003), indicating that host identity significantly shaped root endosphere community assembly across the geographically separated sampling sites. Notably, the bacterial communities in the roots of *T. mirabilis* and its associated soil from the central Thailand sampling site were compositionally distinct from those of the other *Thismia* species and their corresponding soils from southern Thailand, reflecting both the geographic and host-associated divergence in bacterial community structure. Furthermore, pairwise PERMANOVA results indicated that the bacterial communities in *Thismia* roots and soil samples from the diverse ecosystems were significantly different (Supplementary Table S1).

To disentangle the relative contributions of geographic location and host species identity to bacterial community assembly, Mantel and partial Mantel tests were performed separately for soil and root compartments (Table 1). In soil, geographic location was a significant predictor of bacterial community dissimilarity (Mantel r = 0.350, *p* = 0.001), whereas host species showed no significant effect (r = 0.057, *p* = 0.152), indicating that spatial distance primarily structures the soil bacterial community. The opposite pattern was observed in root samples. Geographic location had no significant association with root bacterial community dissimilarity (r = 0.082, *p* = 0.098), while host species identity was a significant predictor (r = 0.176, *p* = 0.002). Partial Mantel tests, which statistically controlled for the confounding variable, corroborated these findings. When host species was controlled, geographic location remained a significant predictor in soil (r = 0.350, *p* = 0.001) but lost significance in roots (r = 0.029, *p* = 0.307). Conversely, when geographic location was controlled, host species identity retained a significant effect on root bacterial communities (r = 0.159, *p* = 0.004) but not on soil communities (r = −0.058, *p* = 0.870). Collectively, these results demonstrate that while geographic location structures the surrounding soil bacterial microbiome, host-mediated selection overrides spatial effects within the *Thismia* root endosphere, driving conserved bacterial community assembly across geographically separated sites.

**Figure 4:**
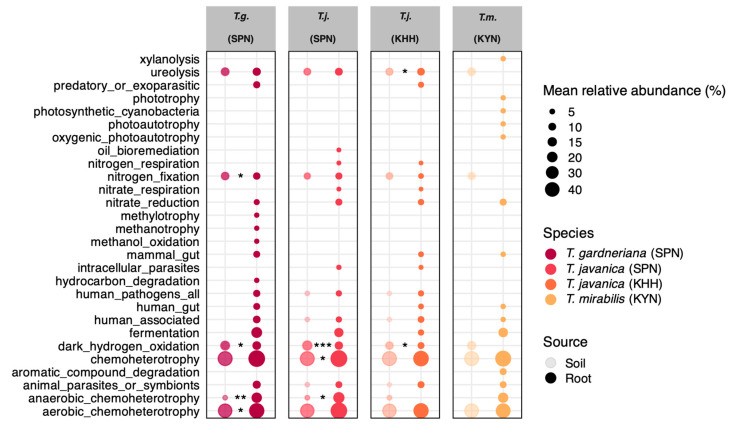
Functional prediction of bacterial communities reveals enrichment of specialized metabolic pathways in the *Thismia* roots. Predicted functional profiles based on 16S rRNA gene sequences using FAPROTAX, showing mean relative abundance of key metabolic pathways across soil and root samples from *T. gardneriana* in Si Phang Nga National Park (SPN) (*n =* 12), *T. javanica* in SPN (*n =* 10) and Ko Hong Hill (KHH) (*n =* 5), and *T. mirabilis* in Khao Yai National Park (KYN) (*n =* 4). Circle size represents the mean relative abundance of each functional category. Different colors indicate species and sampling location, while lighter and darker shades of the same color distinguish soil and root compartments, respectively. Statistical analysis was performed using the Wilcoxon rank sum test with asterisks indicating significant differences (* *p* ≤ 0.05, ** *p* ≤ 0.01, *** *p* ≤ 0.001) between soil and root compartments.

FAPROTAX analysis of predicted bacterial functional profiles revealed distinct metabolic specialization patterns between soil- and root-associated bacterial communities across all three *Thismia* species and geographic locations (Figure 4). Root endosphere communities consistently harbored bacteria with enhanced capacities for nitrogen cycling compared to surrounding soil microbiomes. Nitrate respiration and nitrate reduction pathways were consistently enriched in root samples across all species compared with soil. However, notable differences were observed in *T. mirabilis* relative to *T. gardneriana* and *T. javanica*. Nitrogen fixation, which was enriched in the roots of *T. gardneriana* and *T. javanica*, was absent in *T. mirabilis* roots, suggesting potential interspecific variation in nitrogen acquisition strategies among *Thismia* species.

### 2.3. Root-Associated Bacterial Isolates and Their Phylogenetic Affiliation

A total of 24 bacterial isolates were obtained, all belonging to the phylum Bacillota, specifically the class Bacilli. Of these, two isolates were classified within the order Paenibacillales and 22 within the order Bacillales. All isolates in Paenibacillales belonged to the genus *Paenibacillus*. Within Bacillales, 15 isolates were assigned to the genus *Bacillus*, four to *Gottfriedia*, two to unclassified members of the family Planococcaceae, and one to *Neobacillus*.

**Figure 5:**
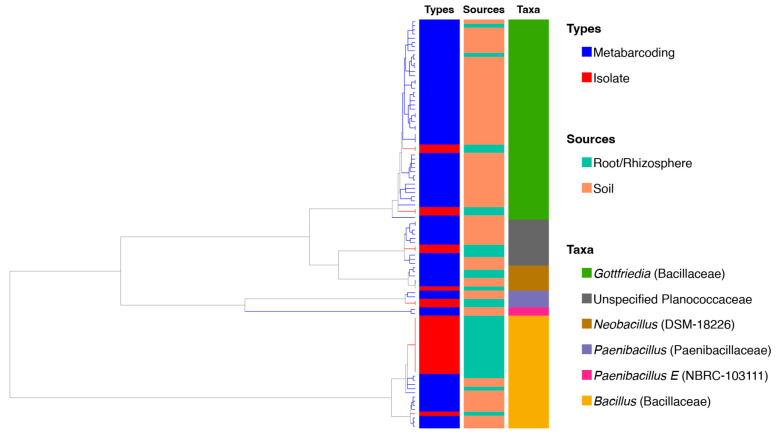
Phylogenetic clustering and taxonomic composition of bacterial isolates and environmental amplicons. The dendrogram illustrates the hierarchical relationships among sequences based on similarity in the 16S rRNA gene. Colored bars indicate sample origins and the corresponding taxonomic affiliations at the genus level. Isolates obtained in this study (red) are distributed within clades dominated by *Bacillus*, *Gottfriedia*, *Paenibacillus*, unclassified Planococcaceae, and *Neobacillus* which are closely affiliated with amplicon sequences from soil and root-associated microbiota.

When incorporated into the phylogenetic tree alongside representative sequences from the metabarcoding dataset, *Paenibacillus* isolates were found to be affiliated with amplicons retrieved from soil samples (Figure 5). Isolates of Bacillus and *Gottfriedia* clustered closely with environmental amplicons derived from soil samples. The two unclassified Planococcaceae isolates also showed close affiliation with soil-derived amplicons. The *Neobacillus* isolate was related to soil sequences but showed its closest association with an amplicon originating from the root of *T. javanica* (Supplementary Figure S2).

A total of 24 bacterial isolates obtained from *T*. *gardneriana* and *T*. *arachnites* were taxonomically classified into five genera: *Bacillus*, *Paenibacillus*, *Lysinibacillus*, *Gottfriedia*, and *Neobacillus* (Supplementary Table S2). Isolates from *T. gardneriana* were distributed among *Bacillus*, *Lysinibacillus*, and *Neobacillus*, whereas the majority of isolates from *T. arachnites* belonged to the genus *Bacillus*.

Functional screening revealed that siderophore production was the most widespread trait among the isolates (Supplementary Table S2 and Supplementary Figure S4). Most strains across all genera exhibited positive siderophore production, including all isolates of *Paenibacillus*, *Lysinibacillus*, *Neobacillus*, and the majority of *Bacillus* and *Gottfriedia*. Only a few isolates, such as TAS1114, TAS1118, and TAS1204, showed no detectable siderophore production.

In contrast, none of the isolates demonstrated phosphate solubilization or the ability to grow on nitrogen-free medium, indicating the absence of detectable phosphate-solubilizing activity and nitrogen fixation potential under the tested conditions (Supplementary Table S2 and Supplementary Figures S5 and S6).

IAA production was observed in a subset of isolates. All isolates from *T. gardneriana* (TGIF2V, TGIF202, and TGR01) tested positive for IAA production, as did several isolates from *T. arachnites*, including *Bacillus* strains (e.g., TAS1106, TAS1109–TAS1111, and TAS1211), *Paenibacillus* (TAS1116), *Lysinibacillus* (TAS1208), and *Gottfriedia* (TAS1212, TAS1214, TAS1215) (Supplementary Table S2 and Supplementary Figure S7). Notably, *Neobacillus* (TGR01) exhibited both siderophore production and IAA synthesis.

Some isolates (e.g., TAS1114, TAS1118, and TAS1204) did not display any of the tested plant growth-promoting traits. Additionally, two isolates (TAS1108 and TAS202) were not evaluated due to lack of growth under the assay conditions.

Overall, these results indicate that siderophore production is the dominant functional trait among the isolates, while IAA production is present in a subset of strains. Other plant growth-promoting traits, including phosphate solubilization and nitrogen fixation, were not detected in this study.

## 3. Discussion

All three *Thismia* species showed consistent enrichment of Pseudomonadota and Bacteroidota in root tissues relative to surrounding soil, despite the 600 km geographic separation between sampling sites (Figure 2a). Notably, Bacteroidota abundance in roots was more than three-fold higher than in the corresponding soil samples across all species, reflecting a conserved pattern of selective bacterial recruitment independent of geographic origin (Figure 2a). This consistency reflects conserved bacterial recruitment across geographically separated *Thismia* species. The enrichment of these two phyla in *Thismia* roots is consistent with a recent study on *T. gardneriana* from the same lowland tropical rainforest in southern Thailand, which reported compartment-specific enrichment of Pseudomonadota and Bacteroidota of the genus *Puia* in the root endosphere [3]. The current multi-species and multi-site analysis extends these findings, demonstrating that this pattern of Pseudomonadota and Bacteroidota enrichment is conserved not only within a single *Thismia* species across seasons, but also across taxonomically distinct *Thismia* species separated by geographic distance.

Plants from geographically distant locations actively employ selective bacterial recruitment strategies to maintain consistent bacterial profiles, suggesting that universal principles govern the plant–microbe associations rather than local environmental factors. This pattern is exemplified by the MHP *Pterospora andromedea,* where Pseudomonadota (formerly Proteobacteria) emerged as the dominant bacterial taxon across five separate sites in the United States. Despite the geographic separation of these locations, the bacterial community structure remained similar, with Pseudomonadota consistently showing higher abundances in the root endosphere than the surrounding bulk soil at each site [7]. This pattern also occurs in photosynthetic plants, as demonstrated in the desert shrub *Tamarix ramosissima*, where Pseudomonadota was the most abundant bacterial group in roots and soil across three different Chinese desert locations, with roots consistently containing higher levels than soil at each site [28]. Furthermore, the relative abundance of Pseudomonadota was consistently enriched in the roots of *Geodorum* terrestrial orchids [29], which preferentially recruited Bacteroidota in their roots [29]. Increased Bacteroidota abundance was also documented in oilseed rape root tissues cultivated under organically managed soil [30]. Conversely, Planctomycetota (formerly Planctomycetes) and Acidobacteriota (formerly Acidobacteria) were uniformly depleted in root tissues across all the three *Thismia* species studied here, confirming the existence of active selection mechanisms (Figure 2a,b). The relative abundance of Acidobacteriota was also depleted in the roots of the *Geodorum* terrestrial orchids [29]. In the present study, the enrichment of Bacteroidota coupled with the depletion of Planctomycetota and Acidobacteriota points to compositional dynamics within the *Thismia* root-associated bacterial community. These findings demonstrate conserved bacterial profiles across the geographically separated *Thismia* species, indicating that these MHPs may employ universal strategies for assembling their root microbiomes.

Root bacterial communities showed significantly reduced diversity compared to soil with lower ASV richness and Shannon diversity but maintained similar evenness, indicating selective filtering rather than random colonization (Figure 3a). In the MHP *P. andromedea*, the bacterial richness and diversity of interior and exterior ectomycorrhizal roots were significantly lower than those in the rhizosphere and bulk soil [7]. Moreover, the bacterial diversity in three different *Geodorum* orchids was consistently lower than in their rhizosphere compartments [29]. Furthermore, the reduction in bacterial richness and diversity in *Thismia* roots observed in this study is consistent with previous findings in autotrophic plants, where root communities typically exhibit lower diversity than surrounding soil environments due to host-mediated selective pressures and niche filtering processes [7,28,29,30]. This study reveals active bacterial filtering and selection by *Thismia* hosts, demonstrating that these MHPs are not passive recipients of their bacterial partners but active curators.

This study found that the host species effect was stronger than geographic distance. The PERMANOVA analysis revealed that *Thismia* roots actively filtered bacterial communities, resulting in root endosphere compositions that were markedly distinct from surrounding soil (Figure 3b) since the root endosphere microbiota is well-established to be primarily assembled from the surrounding bulk soil through a sequential filtering process via the rhizosphere [31,32,33]. Mantel and partial Mantel tests further supported the host species effect, showing that root endosphere community structure was significantly associated with host species identity rather than different geographical habitats (Table 1). This is further reflected by the compositional distinctiveness of *T. mirabilis* roots and its associated soil from the central Thailand sampling site relative to the other *Thismia* species and their corresponding soils from southern Thailand, highlighting that both site-specific differences in the available soil bacterial pool and host identity contribute to shaping the bacterial communities associated with each *Thismia* species. Nevertheless, the pronounced differences between soil and root compartments, particularly the consistent enrichment of Bacteroidota and Pseudomonadota alongside the depletion of Acidobacteriota, Actinomycetota, and Planctomycetota in root samples regardless of location (Figure 2b,c), demonstrate that *Thismia* plants selectively filter and recruit specific bacterial taxa from the local soil pool rather than randomly incorporating taxa from the surrounding soil community.

FAPROTAX analysis predicted consistent enrichment of nitrogen cycling pathways, especially nitrate reduction in the root endosphere across all species and locations (Figure 4). Nitrate reductase (NR) serves as an essential enzyme in plant root systems that converts nitrate into nitrite, initiating the nitrogen assimilation. It enables plants to access nitrate, a primary nitrogen source available in soil. Although NR mainly operates within plant cells, certain bacteria in the endosphere can also participate in nitrate reduction processes [31]. Moreover, it was reported that roots contained more nitrogen-metabolizing bacteria than bulk soil and rhizosphere areas. This was particularly evident for bacterial communities involved in reducing nitrates, using nitrates for respiration, and fixing nitrogen [32]. It should be noted that these are taxon-based functional predictions inferred from 16S rRNA gene sequences and do not represent direct measurements of gene expression or enzymatic activity; future metagenomic or metatranscriptomic studies will be needed to confirm whether these pathways are actively expressed.

Apart from functional predictions, the present study showed that bacteria belonging to Pseudomonadota and Bacteroidota were enriched in the roots of all the *Thismia* collected from the different habitats (Figure 2b,c). In temperate deciduous forests, bacterial communities within leaf litter are notably enriched with Pseudomonadota and Bacteroidota. Moreover, these bacteria are identified as cellulolytic bacteria concomitantly and abundantly found in litter. These microhabitats align with the forest floor environments where *Thismia* are typically found (Figure 1b–d), as these habitats are characterized by substantial accumulations of decomposing leaf litter that would support similar bacterial populations. The enrichment of Pseudomonadota and Bacteroidota in *Thismia* roots may originate from bacterial communities associated with leaf litter, which were not mainly captured by our soil sampling protocol. However, leaf litter was not sampled in the present study, and this suggestion requires further investigation. Future studies incorporating leaf litter as an additional sample compartment alongside bulk soil and root endosphere will be necessary to determine whether litter-associated bacterial communities contribute to the assembly of the *Thismia* root microbiome.

Our findings demonstrate that Bacteroidota, especially the *Puia* genus, as the dominant group was notably enriched in *Thismia* roots (Figure 2c). This is consistent with our previous study on *T. gardneriana* collected during the preceding season, in which Bacteroidota, particularly the genus *Puia,* was similarly identified as the dominant bacterial phylum enriched in the root endosphere, suggesting that this enrichment pattern represents a stable and reproducible feature of the *Thismia* root microbiome assembly across sampling periods [3]. These bacterial species are preferentially enriched in plant roots [33]. Most bacteria within this phylum carry *nif* genes, involved in nitrogen metabolism and can fix nitrogen because they possess the minimum set of genes required for functional nitrogenase [34]. Moreover, these bacteria have adapted to life in the plant microbiome by specializing in available phosphate (Pi) acquisition and play an important role in increasing Pi availability for plants. Most bacterial species typically possess one or more of three broad-spectrum phosphatases, including *PhoA*, *PhoX*, and *PhoD*, which function optimally in alkaline conditions [35]. Furthermore, Lv, et al. (2017) [36] demonstrated that *Puia dinghuensis*, isolated from monsoon evergreen broad-leaved forest soil, produces both alkaline and acid phosphatases, facilitating the conversion of organic phosphorus into bioavailable phosphate for plant uptake. This dual capacity for nitrogen fixation and phosphate mobilization positions these Bacteroidota as essential nutritional partners for *Thismia*, potentially providing critical access to limiting nutrients in the nutrient-poor forest floor environments where these MHP persist.

The isolation of bacterial strains was conducted as a complementary objective of this study to support the culture-independent analyses. A total of 24 isolates were obtained from *Thismia* roots, all of which belonged to the phylum Bacillota. This phylum was also detected in the *Thismia* root microbiome in varying abundances, accounting for up to 20% of the total community (Figure 2a). The dominant classes associated with *Thismia* roots were Clostridia and Bacilli, particularly members of the family Lachnospiraceae (Clostridia) and DSM-18226 (Bacilli), which includes *JAEVLT01*, *Mesobacillus*, and *Neobacillus* (Supplementary Figure S2).

The isolates obtained in this study were exclusively affiliated with the phylum Bacillota. It is important to note that the use of Yeast Mannitol Agar (YEMA), a selective medium designed to favor the growth of *Rhizobiales*, may introduce a cultivation bias and influence the composition of the recovered isolates. Therefore, the predominance of Bacillota observed in this study may reflect selective cultivation effects rather than the true representation of the *Thismia* root microbiome.

Among the isolates, strain TGR01 was identified as belonging to the genus *Neobacillus* (Supplementary Table S2). Members of this genus have been reported from various plant-associated habitats, including *Neobacillus rhizosphaerae* from maize roots and rhizosphere [37], *Neobacillus citreus* from citrus rhizosphere soil [38], *Neobacillus cucumis* from cucumber rhizosphere (*Cucumis sativus*) [39,40], and *Neobacillus endophyticus* from *Selaginella involvens* roots [41,42].

Functional characterization revealed that TGR01 exhibited siderophore production and positive indole-3-acetic acid (IAA) production (Supplementary Table S2 and Supplementary Figures S4 and S7). Similar plant growth-promoting traits were observed in several other isolates in this study, suggesting that members of the isolated Bacillota may contribute to plant–microbe interactions through mechanisms such as iron acquisition and phytohormone production. Previous studies have also reported that *Neobacillus* spp. possess plant growth-promoting capabilities, including the production of IAA, siderophores, proteases, and phosphate solubilization, and can enhance plant growth, such as in *Physalis ixocarpa* [43].

The close association of *Neobacillus* with *Thismia* roots suggests a potentially beneficial role in the plant’s growth or nutrient acquisition. However, experimental validation of this hypothesis is constrained by the scarcity of *Thismia* specimens and the current inability to cultivate the plant under laboratory conditions. Future genomic analyses of the isolated *Neobacillus* strain could provide insights into its biosynthetic potential and plant-associated functional traits, particularly genes involved in IAA and siderophore production, which may elucidate its ecological role in relation to *Thismia*.

## 4. Materials and Methods

### 4.1. Plant and Soil Collection

Three *Thismia* species were observed at three locations in Thailand during 2023. *T. gardneriana* (*n =* 12) and *T. javanica* (*n =* 10) were observed and collected from a lowland tropical rainforest in Si Phang Nga National Park (SPN), Phang Nga Province, every month from May to December 2023 (8°59′56.9″ N 98°27′43.3″ E). *T. mirabilis* (*n =* 4) was observed and collected in Khao Yai National Park (KYN), Nakhon Nayok Province, in June 2023 (14°22′13.1″ N 101°24′33.7″ E). *T. javanica* (*n =* 5) was also observed and sampled on Ko Hong Hill (KHH), Songkhla Province, in August 2023 (7°00′37.1″ N 100°30′42.3″ E). For each *Thismia* root sample collected, a corresponding bulk soil sample was obtained to serve as a paired environmental reference. Prior to soil coring, the surface leaf litter layer was carefully removed. Bulk soil was then collected from a depth of 0–15 cm using sterilized stainless-steel soil corers. Around each *Thismia* individual, three soil subsamples were taken at equidistant positions within a 0.5 m^2^ area and pooled into one composite sample per plant. All soil samples were stored separately in sterile polyethylene bags. Sample sizes (*n*) reported throughout the study represent independent biological replicates, each consisting of a distinct *Thismia* plant individual and its corresponding pooled bulk soil composite. Monthly data for the 10-year period from 2016 to 2025 were obtained from the Thai Meteorological Department. The dataset included average precipitation, relative humidity, and mean temperature for Si Phang Nga National Park (WMO Station ID 561201), Ko Hong Hill (WMO Station ID 568301), and Khao Yai National Park (WMO Station ID 417201). These station records provide the climatic context for interpreting environmental conditions at each study site (Supplementary Figure S1).

### 4.2. DNA Extraction of Root and Soil Samples

The roots of *Thismia* plants were washed with sterile phosphate-buffered saline containing 130 mM NaCl, 7 mM Na_2_HPO_4_, 3 mM NaH_2_PO_4_, and 0.02% (*v*/*v*) Silwet L−77 (pH 7.0). The roots were washed three times for 15 min and then preserved in RNA Protect Tissue Reagent (Qiagen, Hilden, Germany). Genomic DNA was extracted from 50 mg of *Thismia* root, using CTAB as described by Doyle and Doyle (1987) [44]. The extracted DNA sample was suspended in 500 µL of deionized water and purified in the MB spin column of the DNeasy Plant Pro Kit (Qiagen, Hilden, Germany). Soil samples from the habitats were similarly preserved with RNA Protect Tissue Reagent (Qiagen, Hilden, Germany), and genomic DNA was extracted using the DNeasy PowerSoil Pro Kit (Qiagen, Hilden, Germany). All DNA samples were stored at −20 °C until further processing.

### 4.3. Analysis of 16S Amplicon Sequences

The V3–V4 region of the 16S rRNA gene was amplified using the forward primer 338F (5′-ACTCCTACGGGAGGCAGCA-3′) and the reverse primer 806R (5′-GGACTACHVGGGTWTCTAAT-3′) [45]. Extraction blanks and PCR blanks were included as negative controls. Gel electrophoresis of all negative controls confirmed the absence of PCR amplification products, indicating no contamination was introduced during DNA extraction or PCR amplification. Illumina MiSeq amplicon paired-end sequencing was conducted at BMK GENE (Hong Kong, China). The average sequencing depth was 99,379 reads per sample (range: 33,152–254,955). The demultiplexed raw sequence data were trimmed to the V3–V4 region using uclust. Amplicon sequence variants (ASVs) were identified using DADA2 [46] via the q2-dada2 plugin, with truncation lengths set to 250 bp. Default denoising parameters were applied (maximum expected errors = 2.0), and non-overlapping read pairs were discarded. ASVs with a total abundance below 14,000 reads across all samples were filtered out prior to taxonomic classification. Rare ASVs with a total frequency of fewer than 10 reads across all samples were removed. Bacterial community bioinformatics was performed using the Silva Database (SILVA 138.2). Subsequently, mitochondrial and chloroplast sequences were filtered out, and the remaining sequences were classified with the Genome Taxonomy Database (GTDB Release 220) [47]. The data were then imported into Phyloseq [48] in R version 4.5.1 [49]. The data for this study have been deposited in the European Nucleotide Archive (ENA) at EMBL-EBI under accession number PRJEB101102.

### 4.4. Analysis of Bacterial Microbiome

Maps of sample site locations were plotted using the packages sf [50], rnaturalearth [51], and rnaturalearthdata [52]. Relative abundances were presented in stack bar plots. Differential abundance analysis was conducted using ANCOM-BC [53] implemented in QIIME 2 to identify taxa exhibiting significant differences in relative abundance among soil, and endosphere compartments. Analyses were performed at the phylum and genus levels. Taxa with adjusted *p*-values below the specified significance threshold (*p* < 0.001) were considered differentially abundant between the compartments. Alpha diversity metrics including observed richness, evenness, and Shannon diversity were analyzed. Means were compared using the Wilcoxon rank sum test. Beta diversity was determined from clustering, analyzed by Principal Coordinate Analysis (PCoA) based on Bray–Curtis dissimilarity using the vegan package version 2.7-3 in R [54]. Comparisons of differential bacterial compositions across plant species and compartments were analyzed by Permutational Multivariate Analysis of Variance (PERMANOVA) based on Bray–Curtis dissimilarity, using 999 permutations with the vegan package in R. Mantel and partial Mantel tests were performed independently for root and soil compartments. Bacterial community dissimilarity was calculated using Bray–Curtis dissimilarity, and geographic and host species distance matrices were generated using Euclidean distances. Statistical significance was evaluated using 9999 permutations with Pearson’s correlation coefficient, using the mantel and mantel partial functions from the vegan package in R. Functional predictions of bacteria found in endosphere and soil samples were performed using the FAPROTAX database in the microeco package version 2.1.0 in R [55]. Differences in predicted functional pathway abundances between root and soil within the same *Thismia* species and location compartments were assessed using the Wilcoxon rank-sum test in R. All visualizations—including stacked bar plots for relative abundances, box plots for alpha diversity and 10-year climate data, PCoA scatter plots for beta diversity, horizontal bar plots for ANCOM-BC log fold changes, and dot plots for functional profiling—were created with ggplot2 version 4.0.2 [56] in R.

### 4.5. Culture-Dependent Analysis of Root-Associated Bacteria

#### 4.5.1. Isolation of Root-Associated Bacteria

Bacterial isolation was carried out to obtain cultivable root-associated bacteria from *Thismia* root tissue for functional characterization. The roots of *T. gardneriana* and *T. arachnites* were gently washed multiple times with sterile distilled water to remove adhering soil particles until visibly clean. *T. arachnites* was not included in the amplicon sequencing analysis due to limited sample biomass. Afterward, the roots were surface-sterilized by soaking in 70% (*v*/*v*) Ethanol for 30 s, then rinsed with sterile distilled water for another 30 s. Next, the roots were soaked in 2% (*v*/*v*) NaOCl for 60 s, followed immediately by immersion in 95% (*v*/*v*) Ethanol. The remaining residues on the roots were removed by submerging the roots in sterile distilled water for 30 s. The surface-sterilized roots were allowed to dry in the biosafety cabinet.

The roots were aseptically crushed using a sterile spatula. A small volume of sterile distilled water was added, and the resulting suspension was streaked onto Yeast Mannitol Agar (YEMA) medium (10.0 g/L mannitol, 0.5 g/L K_2_HPO_4_, 0.5 g/L yeast extract, 0.2 g/L MgSO_4_·7H_2_O, 0.1 g/L NaCl, and 1.5% (*w*/*v*) agar). YEMA medium was selected to preferentially isolate members of *Rhizobium*, which have been previously detected in *Thismia* root microbiomes and may play roles in plant–microbe interactions [3]. The plates were incubated at ambient temperature for 3–5 days. Emerging colonies were repeatedly streaked on YEMA plates to obtain pure isolates.

#### 4.5.2. Identification and Phylogenetic Analyses of Bacterial Isolates

The colony PCR was performed using the AllTaq PCR Core Kit (QIAGEN, Hilden, Germany) following the manufacturer’s instructions. The 16S rRNA gene was amplified with universal primers 27F (5′-AGAGTTTGATCCTGGCTCAG-3′) and 1492R (5′-GGTTACCTTGTTACGACTT-3′) to identify the isolated bacteria. The PCR conditions included an initial denaturation at 95 °C for 5 min, followed by 30 cycles of 95 °C for 1 min, 55 °C for 1 min, 72 °C for 1 min, and a final extension at 72 °C for 5 min. The amplified PCR products were sent for sequencing at Celemics, Seoul, Republic of Korea. The sequences were deposited under GenBank accession numbers PX446825-PX446848.

The 16S rRNA gene sequences were processed using QIIME2 version 2024.10. Raw sequences were trimmed to the targeted amplicon region defined by the primers 338F (5′-ACTCCTACGGGAGGCAGCA-3′) and 806R (5′-GGACTACHVGGGTWTCTAAT-3′) used for library preparation. The trimmed reads were subsequently merged to obtain representative sequences for downstream analyses. Taxonomic assignment was performed using the same pre-trained classifier employed in the metabarcoding dataset to ensure consistency in annotation between culture-independent and culture-dependent approaches.

To assess the phylogenetic relationship between the isolates and the microbial community identified through metabarcoding, a rooted phylogenetic tree was constructed using the QIIME2 pipeline qiime phylogeny align-to-tree-mafft-fasttree, which includes sequence alignment with MAFFT, masking of hypervariable regions, and tree inference with FastTree. Version 2.1.11. The clades containing the isolates were extracted and visualized using the q2-Empress plugin [57], enabling direct comparison of cultured isolates within the broader community context.

#### 4.5.3. Characterization of Plant Growth-Promoting Traits of Bacterial Isolates

Plant growth-promoting (PGP) traits, including siderophore production, phosphate solubilization, nitrogen fixation, and indole-3-acetic acid (IAA) production, were evaluated to assess the functional potential of the isolated bacteria. All assays were performed following Balagurunathan, et al. (2020) [58] with modifications as described below.

Bacterial isolates were first grown on Yeast Mannitol Agar (YEMA). For siderophore production, agar plugs of actively growing cultures were inverted onto Chrome Azurol S (CAS) agar. CAS agar was prepared using Chrome Azurol S (60.5 mg in 50 mL distilled water), hexadecyltrimethylammonium bromide (72.9 mg in 40 mL distilled water), and King’s B medium base (20 g/L proteose peptone, 1.5 g/L K_2_HPO_4_, 1.5 g/L MgSO_4_·7H_2_O, 15 mL of glycerol and 20 g/L agar) adjusted to pH 6.8 ± 0.2. Siderophore production was indicated by the formation of an orange halo surrounding the colony.

For phosphate solubilization, agar plugs were placed on Pikovskaya’s (PVK) agar containing 0.5 g/L yeast extract, 10 g/L dextrose, 5 g/L Ca_3_(PO_4_)_2_, 0.5 g/L (NH_4_)_2_SO_4_, 0.2 g/L KCl, 0.1 g/L MgSO_4_, 0.0001 g/L MnSO_4_·H_2_O, 0.0001 g/L FeSO_4_, and 15 g/L agar. Phosphate solubilization was indicated by the formation of a clear halo zone surrounding the colony.

For nitrogen fixation, isolates were streaked onto Jensen’s nitrogen-free agar composed of 20 g/L sucrose, 1 g/L K_2_HPO_4_, 0.5 g/L MgSO_4_, 0.5 g/L NaCl, 0.1 g/L FeSO_4_, 0.005 g/L Na_2_MoO_4_·2H_2_O, 2 g/L CaCO_3_ and 15 g/L agar. Growth along the streak line was interpreted as the ability to grow under nitrogen-free conditions.

For IAA production, isolates were inoculated into ISP2 broth (4.0 g/L yeast extract, 10.0 g/L malt extract, 4.0 g/L dextrose, pH 7.2) supplemented with L-tryptophan (2 g/L). A negative control consisting of the same medium without tryptophan was included. Cultures were incubated at 30 °C for 7 days in the dark with shaking at 150 rpm. After incubation, cultures were centrifuged at 12,000 rpm for 5 min, and 1 mL of supernatant was mixed with 2 mL of Salkowski reagent and incubated in the dark for 30 min. The development of a pink to red coloration indicated IAA production. All plate-based assays were incubated at 30 °C for 7 days.

## 5. Conclusions

Our study provides the first comprehensive characterization of endophytic bacterial communities in MHP *Thismia* species, revealing a previously unrecognized dimension of their nutritional ecology. Through comparative analysis of three species across a 600 km geographic gradient in Thailand, we demonstrated that these achlorophyllous plants maintain remarkably conserved bacterial partnerships that transcend both taxonomic and spatial boundaries. The consistent enrichment of Pseudomonadota and Bacteroidota, particularly the *Puia* genus, in *Thismia* root endospheres, coupled with the reduced compositional dynamics of Acidobacteriota and Planctomycetota, indicates sophisticated host-mediated selection mechanisms operating across all three species. Our PERMANOVA analysis revealed that bacterial communities in *Thismia* roots were markedly distinct from those in surrounding soil, while root endosphere communities from geographically distinct habitats clustered together regardless of their 600 km separation. Mantel and partial Mantel tests further corroborated this pattern, demonstrating that host species identity was a significant predictor of root bacterial community structure, whereas geographic distance showed no significant association after controlling for host identity, confirming that *Thismia* species employ universal strategies for assembling their root bacterial microbiomes. Functional prediction analyses suggested that root-associated bacterial communities were significantly enriched for nitrogen cycling pathways, particularly nitrogen fixation and nitrate reduction, compared to surrounding soil communities. The selective enrichment of Bacteroidota, known for nitrogen fixation and phosphate mobilization capabilities, suggests these bacteria might provide critical nutritional support in the nutrient-poor forest floor environments where *Thismia* persists. Given the extreme morphological reduction of *Thismia* root systems, these bacterial partnerships likely represent key adaptive strategies for nutrient acquisition. Furthermore, we successfully isolated aerobic bacterial strains from *Thismia* roots, all belonging to Bacillota, including *Neobacillus*, a genus documented for plant growth-promoting traits. These isolates represent valuable resources for future functional studies on plant–bacterial interactions in MHP systems. This research expands our understanding of mycoheterotrophy beyond traditional plant–fungal partnerships and establishes bacterial endophytes as important components of *Thismia* nutritional ecology, warranting further investigation into the mechanisms underlying these associations.

## Figures and Tables

**Table 1:** Mantel and Partial Mantel tests examining the relationships between bacterial community dissimilarity and geographic distance or host species identity in root and soil compartments of three *Thismia* species across geographically distinct sites in Thailand.

Test	Predictor Variable	Compartment	r	*p*-Value	Sig
Mantel	Geographic location	Soil	0.350	0.001	**
	Geographic location	Root	0.082	0.098	ns
	Host species	Soil	0.057	0.152	ns
	Host species	Root	0.176	0.002	**
Partial Mantel	Geographic location|host species	Soil	0.350	0.001	**
	Geographic location|host species	Root	0.029	0.307	ns
	Host species|geographic location	Soil	−0.058	0.870	ns
	Host species|geographic location	Root	0.159	0.004	**

The response variable for all tests was bacterial community dissimilarity based on the Bray–Curtis dissimilarity matrix calculated from 16S rRNA gene amplicon sequencing data. r = Mantel statistic; *p*-values based on 999 permutations. Significance codes: ** *p* < 0.01, ns = not significant.

## Data Availability

The sequence data presented in this study are openly available in GenBank under accession numbers PX446825–PX446848. Other data supporting the findings of this study are available within the article and Supplementary Materials.

## Supplementary Materials

The following supporting information can be downloaded at: https://www.mdpi.com/article/10.3390/plants15091316/s1, Figure S1: Monthly climate patterns across three study sites in Thailand over 10 years (2016–2025); Figure S2: Relative abundance of the bacterial family DSM-18226 and its constituent genera across *Thismia* root and soil samples; Figure S3: Principal coordinate analysis (PCoA) of bacterial community composition across *Thismia* species and study sites; Figure S4: Qualitative screening of siderophore production by bacterial isolates; Figure S5: Qualitative screening of phosphate solubilization by bacterial isolates; Figure S6: Qualitative assessment of nitrogen fixation by bacterial isolates; Figure S7: Qualitative detection of indole-3-acetic acid (IAA) production by bacterial isolates; Table S1: Pairwise PERMANOVA comparisons of bacterial community composition; Table S2: Plant growth-promoting traits of isolated strains.
