# Supplementary material for: Host-Mediated Selection Shapes Conserved Root Bacterial Microbiomes Across Geographically Separated Thismia Species
Source: Plants (Basel). 2026 Apr 25;15(9):1316. doi: 10.3390/plants15091316 (PMC13165429; doi:10.3390/plants15091316)
Supplement: Supplementary file 1 [file plants-15-01316-s001.zip › plants-4266117-supplementary.pdf]

Supplementary Material

Supplementary Figures

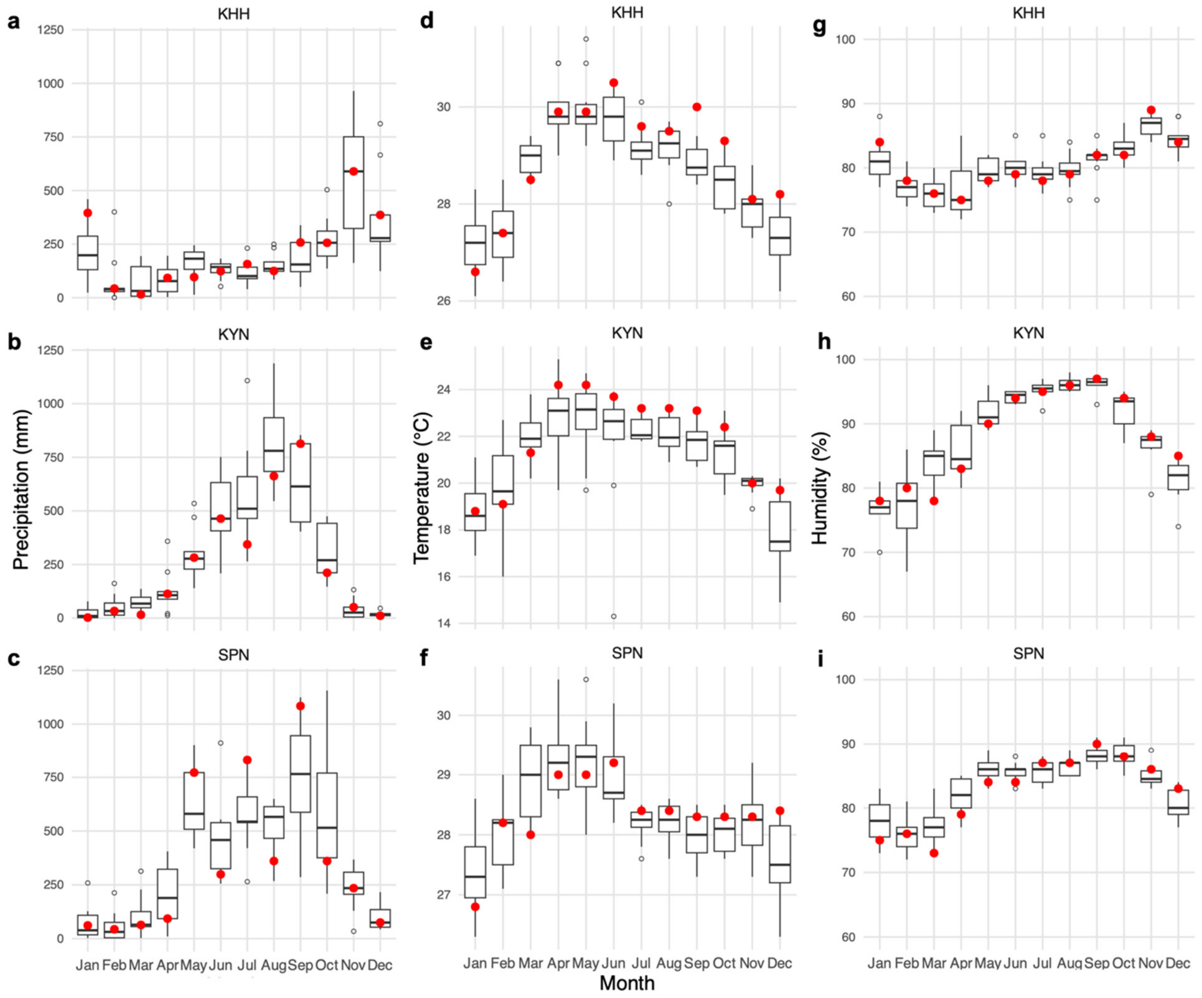

Supplementary Figure S1. Monthly climate patterns across three study sites in Thailand over 10 years (2016-2025). Box plots display the distribution of total precipitation (a-c), average temperature (d-f), and relative humidity (g-i) for Ko Hong Hill, Songkhla, Thailand (The first row); Khao Yai National Park, Nakhon Nayok, Thailand (The second row); and Sri Phang Nga National Park, Phang Nga, Thailand (The third row). Each box represents the interquartile range with median values, while whiskers extend to the full data range, excluding outliers. Red dots highlight data from the focal study year (2023).

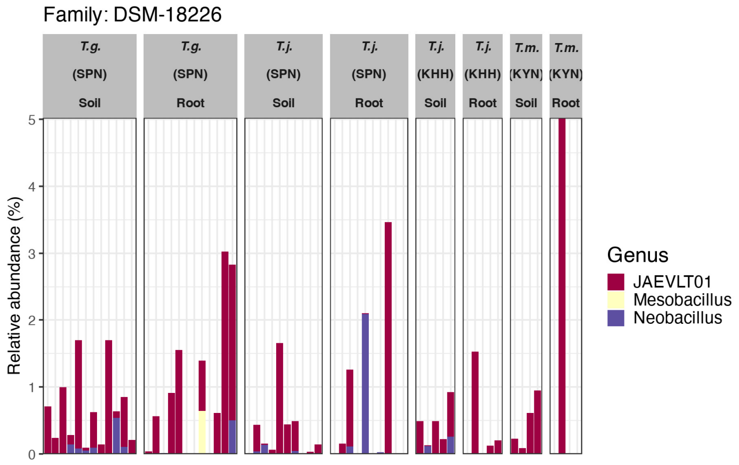

Supplementary Figure S2. Relative abundance of the bacterial family DSM-18226 and its constituent genera across *Thismia* root and soil samples. The bar plot shows the proportional representation of the genera *JAEVLT01*, *Mesobacillus*, and *Neobacillus* within the family DSM-18226 in different *Thismia* species and collection sites: *T. gardneriana* (SPN) (*n* = *12*), *T. javanica* (SPN and KHH) (*n* = *10* and *n* = *5*, respectively), and *T. mirabilis* (KYN) (*n* = *4*). The data indicate that members of DSM-18226 are present in both soil and root-associated microbiomes, with variation in genus-level composition among *Thismia* species and locations.

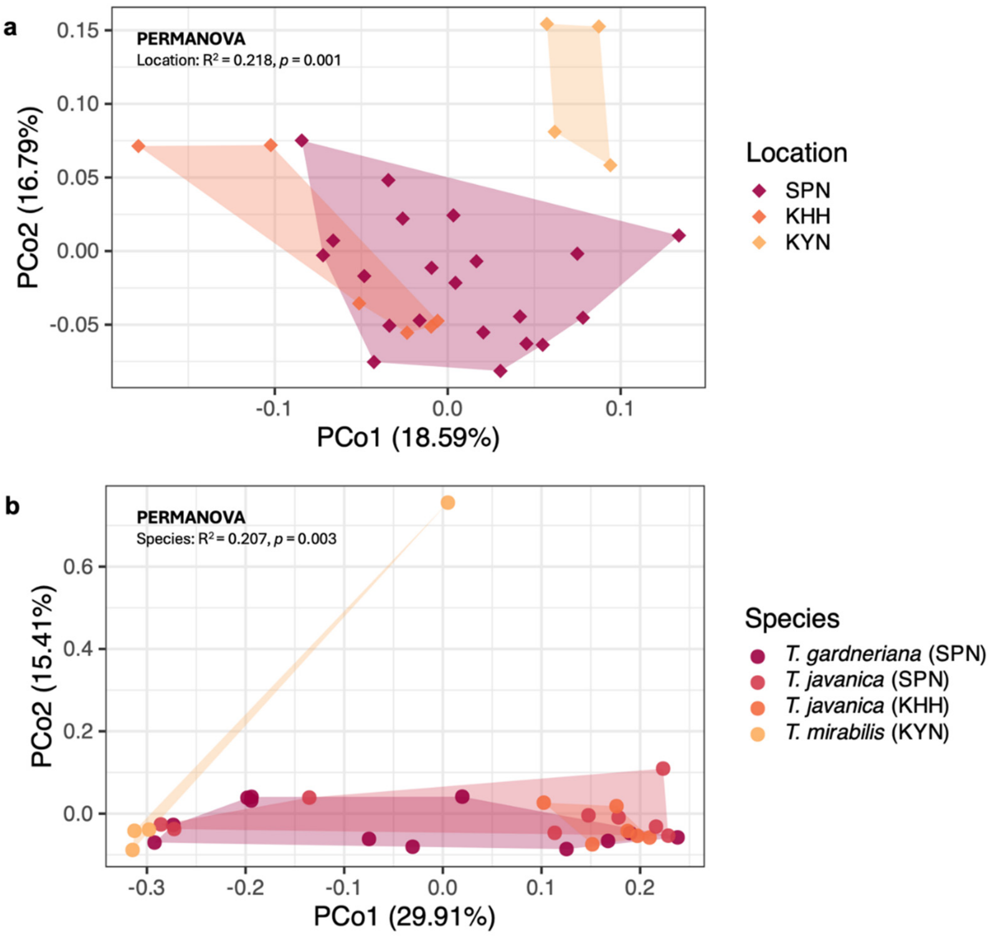

Supplementary Figure S3. Principal coordinate analysis (PCoA) of bacterial community composition across *Thismia species* and study sites. PCoA based on Bray-Curtis dissimilarity illustrates the structure of bacterial communities colored by study site (SPN, Sri Phang Nga National Park, n = 22; KHH, Ko Hong Hill, n = 5; KYN, Khao Yai National Park, n = 4) (a) and by host species (*T. gardneriana* (SPN, n = 12), *T. javanica* (SPN, n = 10), *T. javanica* (KHH, n = 5), and *T. mirabilis* (KYN, n = 4)) (b).

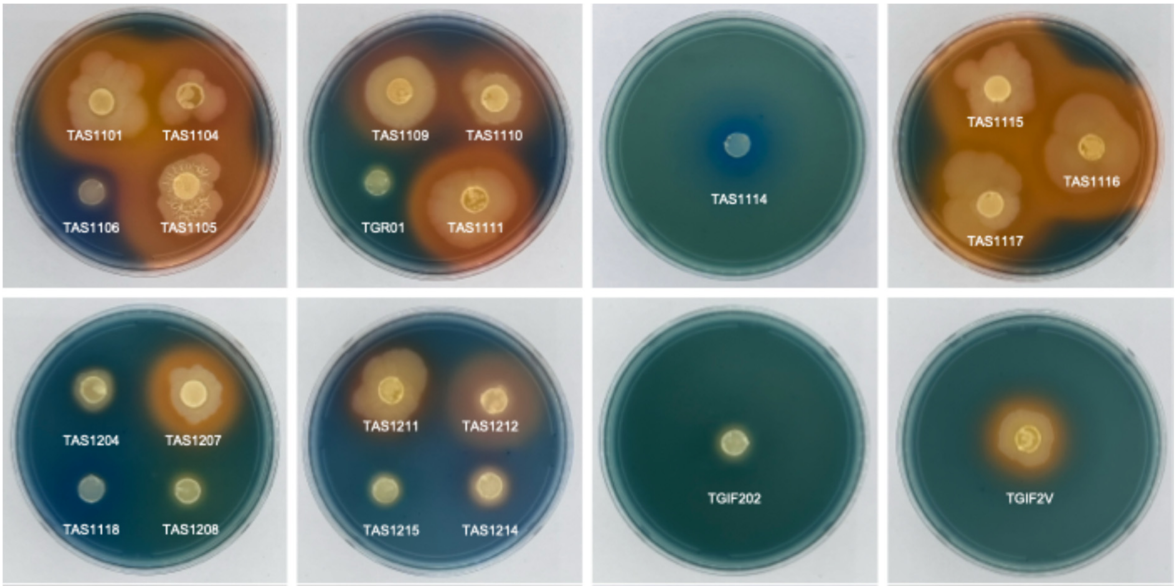

Supplementary Figure S4. Qualitative screening of siderophore production by bacterial isolates on Chrome Azurol S (CAS) agar. Orange halos surrounding bacterial colonies indicate siderophore production, whereas the absence of color change (blue-green medium) indicates negative activity. Most isolates exhibited clear siderophore production with varying halo intensities, while a few strains (e.g., TAS1114) showed no detectable activity. Weak producers displayed smaller or less intense halos compared to strong producers.

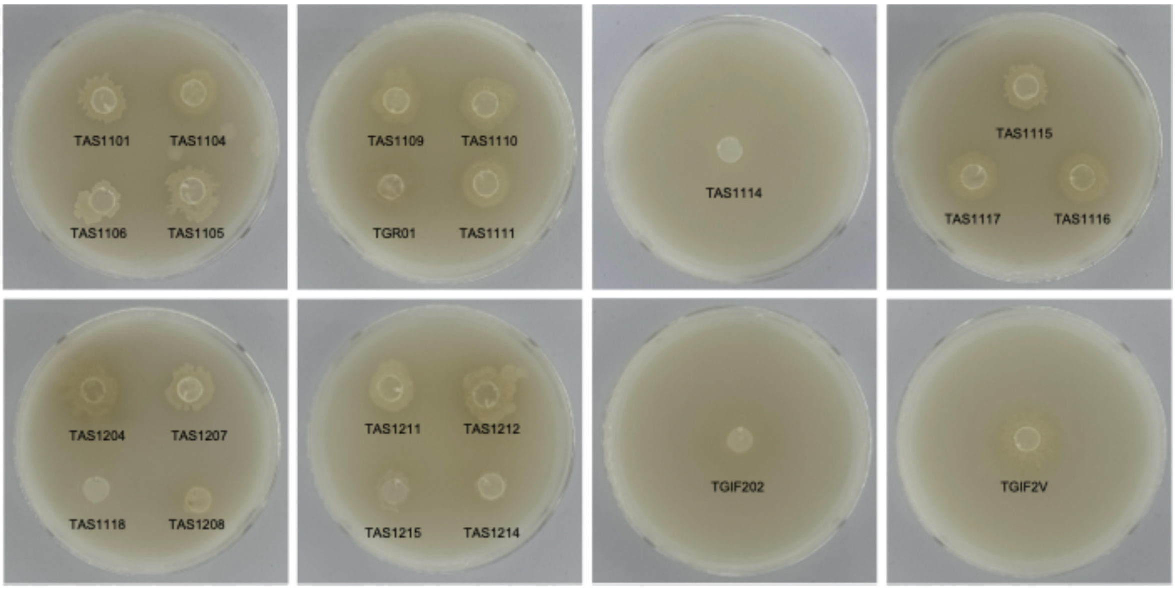

Supplementary Figure S5. Qualitative screening of phosphate solubilization by bacterial isolates on Pikovskaya's agar. The formation of clear halos surrounding bacterial colonies indicates phosphate solubilization, whereas the absence of halo formation suggests negative activity. No visible halo zones were observed around any of the tested isolates, indicating that none exhibited detectable phosphate-solubilizing ability under the conditions tested.

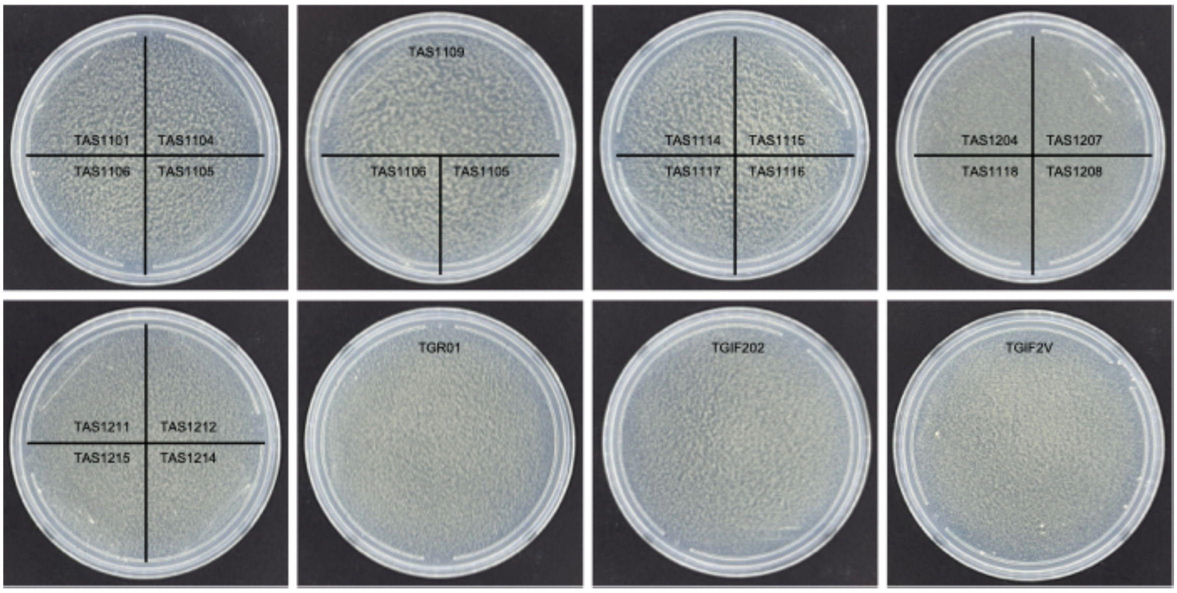

Supplementary Figure S6. Qualitative assessment of nitrogen fixation by bacterial isolates on nitrogen-free medium. The absence of visible colony growth indicates a lack of nitrogen-fixing ability under the tested conditions. No growth was observed for any of the isolates, suggesting that none of the strains were capable of fixing atmospheric nitrogen or sustaining growth in nitrogen-free medium.

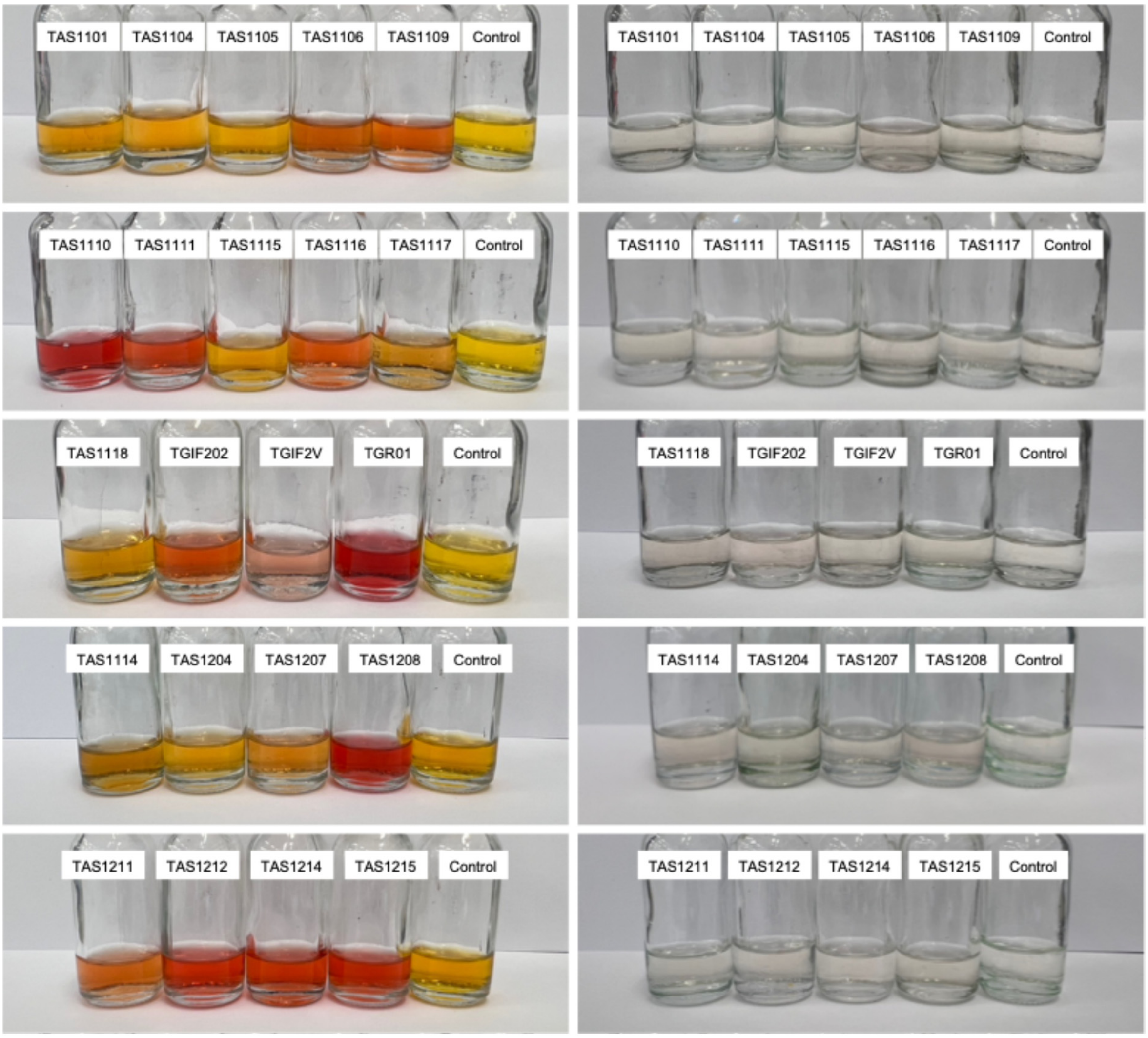

Supplementary Figure S7. Qualitative detection of indole-3-acetic acid (IAA) production by bacterial isolates using Salkowski reagent. Development of pink to red coloration indicates IAA production, whereas yellow or no color change indicates negative results. Several isolates exhibited positive IAA production with varying intensities of red coloration (e.g., TAS1106, TAS1110–TAS1111, TGIF202, TGIF2V, TGR01, TAS1208, and TAS1211–TAS1215), while others showed weak or no detectable activity (Left). Controls without addition of tryptophan remained colorless to pale yellow, confirming the specificity of the reaction (Right).

Supplementary Table

Supplementary Table S1 Pairwise PERMANOVA comparisons of bacterial community composition between *Thismia* species, sampling sites, and compartments. Statistical analysis based on Bray-Curtis dissimilarity with p-values adjusted using the Benjamini-Hochberg (BH) method for multiple comparisons. Sampling sites: SPN, Sri Phang Nga; KHH, Ko Hong; KYN, Khao Yai National Park. ns, non-significant; * $p \leq 0.05$; ** $p \leq 0.01$; *** $p \leq 0.001$.

Compartments	Comparison	R^2	p.value	p.adjusted	sig
**Root compartments**	*T. gardneriana*-root (SPN) vs *T. javanica*-root (KHH)	0.065	0.001	0.004	**
	T. gardneriana-root (SPN) vs *T. javanica*-root (SPN)	0.051	0.102	0.110	ns
	T. gardneriana-root (SPN) vs *T. mirabilis*-root (KYN)	0.068	0.124	0.129	ns
	T. javanica-root (KHH) vs *T. javanica*-root (SPN)	0.075	0.032	0.041	*
	T. javanica-root (KHH) vs *T. mirabilis*-root (KYN)	0.121	0.035	0.043	*
	T. javanica-root (SPN) vs *T. mirabilis*-root (KYN)	0.085	0.096	0.110	ns
**Soil compartments**	*T. gardneriana*-soil (SPN) vs *T. javanica*-soil (KHH)	0.061	0.098	0.110	ns
	T. gardneriana-soil (SPN) vs *T. javanica*-soil (SPN)	0.049	0.813	0.813	ns
	T. gardneriana-soil (SPN) vs *T. mirabilis*-soil (KYN)	0.073	0.017	0.026	*
	T. javanica-soil (KHH) vs *T. javanica*-soil (SPN)	0.078	0.025	0.035	*
	T. javanica-soil (KHH) vs *T. mirabilis*-soil (KYN)	0.118	0.025	0.035	*
	T. javanica-soil (SPN) vs *T. mirabilis*-soil (KYN)	0.094	0.008	0.013	*
**Root and soil compartments**	*T. gardneriana*-root (SPN) vs *T. gardneriana*-soil (SPN)	0.059	0.001	0.004	***
	T. javanica-root (KHH) vs *T. javanica*-soil (KHH)	0.067	0.002	0.004	**
	T. javanica-root (SPN) vs *T. javanica*-soil (SPN)	0.067	0.001	0.004	***
	T. mirabilis-root (KYN) vs *T. mirabilis*-soil (KYN)	0.075	0.027	0.036	*

Supplementary Table S2 Plant growth-promoting traits of isolated strains

Host	Bacterial genera	Strain	Siderophore production	Phosphate solubilization	Nitrogen fixation	IAA production
T. gardneriana	*Bacillus*	TGIF2V	+	−	−	+
	Lysinibacillus	TGIF202	+	−	−	+
	Neobacillus	TGR01	+	−	−	+
T. aracnites	*Bacillus*	TAS1104	+	−	−	−
		TAS1105	+	−	−	−
		TAS1106	+	−	−	+
		TAS1108	NA	NA	NA	NA
		TAS1109	+	−	−	+
		TAS1110	+	−	−	+
		TAS1111	+	−	−	+
		TAS1114	−	−	−	−
		TAS1115	+	−	−	−
		TAS1117	+	−	−	−
		TAS1118	−	−	−	−
		TAS1207	+	−	−	−
		TAS1211	+	−	−	+
		TAS202	NA	NA	NA	NA
	Paenibacillus	TAS1101	+	−	−	−
		TAS1116	+	−	−	+
	Lysinibacillus	TAS1208	+	−	−	+
	Gottfriedia	TAS1204	−	−	−	−
		TAS1212	+	−	−	+
		TAS1214	+	−	−	+
		TAS1215	+	−	−	+

+, activity; –, no activity; N/A, no growth.
